# Molecular Biomarkers in Drug-Induced Liver Injury: Challenges and Future Perspectives

**DOI:** 10.3389/fphar.2019.01667

**Published:** 2020-01-30

**Authors:** Siyu Fu, Dongbo Wu, Wei Jiang, Juan Li, Jiang Long, Chengyao Jia, Taoyou Zhou

**Affiliations:** ^1^Center of Infectious Diseases, West China Hospital, Sichuan University, Chengdu, China; ^2^Department of Infectious Diseases, Pidu District People's Hospital, Chengdu, China; ^3^The Mental Health Center and the Psychiatric Laboratory, West China Hospital, Sichuan University, Chengdu, China; ^4^Department of Laboratory Medicine, West China Hospital, Sichuan University, Chengdu, China

**Keywords:** drug-induced liver injury, biomarkers, scoring systems, hepatotoxicity, diagnosis

## Abstract

Drug-induced liver injury (DILI) is one among the common adverse drug reactions and the leading causes of drug development attritions, black box warnings, and post-marketing withdrawals. Despite having relatively low clinical incidence, its potentially severe adverse events should be considered in the individual patients due to the high risk of acute liver failure. Although traditional liver parameters have been applied to the diagnosis of DILI, the lack of specific and sensitive biomarkers poses a major limitation, and thus accurate prediction of the subsequent clinical course remains a significant challenge. These drawbacks prompt the investigation and discovery of more effective biomarkers, which could lead to early detection of DILI, and improve its diagnosis and prognosis. Novel promising biomarkers include glutamate dehydrogenase, keratin 18, sorbitol dehydrogenase, glutathione S-transferase, bile acids, cytochrome P450, osteopontin, high mobility group box-1 protein, fatty acid binding protein 1, cadherin 5, miR-122, genetic testing, and omics technologies, among others. Furthermore, several clinical scoring systems have gradually emerged for the diagnosis of DILI including the Roussel Uclaf Causality Assessment Method (RUCAM), Clinical Diagnostic Scale (CDS), and Digestive Disease Week Japan (DDW-J) systems. However, currently their predictive value is limited with certain inherent deficiencies. Thus, perhaps the greatest benefit would be achieved by simultaneously combining the scoring systems and those biomarkers. Herein, we summarized the recent research progress on molecular biomarkers for DILI to improved approaches for its diagnosis and clinical management.

## Introduction

Drug-induced liver injury (DILI) remains one of the most challenging diseases to treat by physicians and can be caused by many types of prescription or over-the-counter drugs, biological agents, natural medicines, herbs, dietary supplements, health care products, and their metabolic products or accessories (European Association for the Study of the Liver. Electronic address et al., 2019). Many different drugs have been found to induce liver injury, including herbal and dietary supplements (polygonum multiflorum, camellia sinensis, lycopodium serratum, ephedra, sho-saiko-to, dai-saiko-to, ganoderma lucidum, hydroxycut^®^, dysosma pleiantha, etc.), anti-infectious agents (isoniazid, amoxicillin–clavulanate, minocycline, nitrofurantoin, sulfonamides, azithromycin, ciprofloxacin, levofloxacin, cefazolin, etc.), antineoplastics or immunomodulators (thioguanine, lapatinib, pazopanib, gemtuzumab, interferon beta, busulfan, floxuridine, flutamide, infliximab, etc.), and non-steroidal anti-inflammatory drugs (acetaminophen, diclofenac, lumiracoxib, ibuprofen, naproxen, aspirin, etc.), etc. (European Association for the Study of the Liver. Electronic address et al., 2019; Hoofnagle and Bjornsson, 2019; Shen et al., 2019; Tu et al., 2019). The mechanism underlying DILI has not been fully elucidated, and recent studies suggested that the reactive metabolites of drugs in the liver could generate a variety of biochemical consequences, including covalent binding, stress kinase activation, mitochondria stress, and endoplasmic reticulum stress, which either lead to necrosis or apoptosis or elicit an adaptive immune response to drug-adducts in susceptible individuals (European Association for the Study of the Liver. Electronic address et al., 2019). Further, the difficulty in diagnosing and managing DILI is compounded by the large number of pathogenic drugs on the market, the associated atypical clinical symptoms, and lacking specific and sensitive diagnostic testing. Furthermore, it is hard to estimate the absolute incidence of DILI because of the large populations and prevalence of unregulated drug use in many countries. A study from the state of Delaware in the United States reported DILI-related morbidity was 2.7 cases per 100,000 individuals, which is lower than the incidence of 14–19 per 100,000 inhabitants in France and Iceland (Sgro et al., 2002; Bjornsson et al., 2013; Vega et al., 2017). Recently, a retrospective study reported that 25,927 patients suffering from DILI were hospitalized at 308 medical centers in mainland China. The annual incidence of DILI in the general Chinese population was estimated to be 23.80 per 100,000 inhabitants (Shen et al., 2019). Additionally, it has been reported that the leading implicated drugs were traditional Chinese medicines (TCM) or herbal and dietary supplements (26.81%), antituberculosis medications (21.99%), and antineoplastics or immunomodulators (8.34%) (Shen et al., 2019).

DILI is one of the main causes of chronic liver diseases. It is generally classified as intrinsic (or direct) or idiosyncratic; meanwhile indirect injury is becoming a third type (Hoofnagle and Bjornsson, 2019). Intrinsic DILI is generally dose-dependent and predictable, and occurs in the majority of the individuals exposed to the drug within a short time period (usually with an onset within 1 to 5 days), and can be reproduced in animal models (Hoofnagle and Bjornsson, 2019). Acetaminophen-induced liver injury is a common form of intrinsic DILI, which causes mitochondrial dysfunction and centrilobular necrosis of hepatocytes (McGill and Jaeschke, 2013; European Association for the Study of the Liver. Electronic address et al., 2019). Acute hepatic necrosis is the most common form of intrinsic DILI (Hoofnagle and Bjornsson, 2019). For idiosyncratic DILI, the characteristics are the opposite to those of intrinsic DILI. They generally are not dose-dependent and affect only a small proportion of exposed individuals (European Association for the Study of the Liver. Electronic address et al., 2019; Hoofnagle and Bjornsson, 2019). Acute hepatocellular hepatitis is the most common form of idiosyncratic hepatotoxicity (Andrade et al., 2005; Bjornsson et al., 2013; Chalasani et al., 2015). Although idiosyncratic DILI is rare, it carries with it a higher risk of acute liver failure (ALF), as it is nearly impossible to predict its incubation periods, which can range from weeks to months (McGill et al., 2012; Chalasani et al., 2015). Polygonum multiflorum-induced liver injury (PM-IDILI) is a typical example of idiosyncratic DILI. Mild immune stress (MIS) may be an important mechanism mediating the susceptibility to PM-IDILI by upregulating the levels of chemokines and metabolic reprogramming induced by MIS (Tu et al., 2019). Moreover, recently, a genome-wide association study identified an association between rs2476601 in nonreceptor type 22 gene (PTPN22) and an increased risk of developing idiosyncratic DILI (Cirulli et al., 2019). Finally, indirect DILI is a new and not yet fully accepted category of hepatotoxicity, which results from the action of the drug rather than from its inherent hepatotoxic effects or immunogenicity. It can manifest as a new liver disease or as the deterioration of preexisting conditions (Hoofnagle and Bjornsson, 2019). Antineoplastic agents, glucocorticoids, monoclonal antibodies (against tumor necrosis factor, CD20, or checkpoint proteins), and protein kinase inhibitors were associated with indirect hepatotoxicity (Hoofnagle and Bjornsson, 2019).

**Figure 1:**
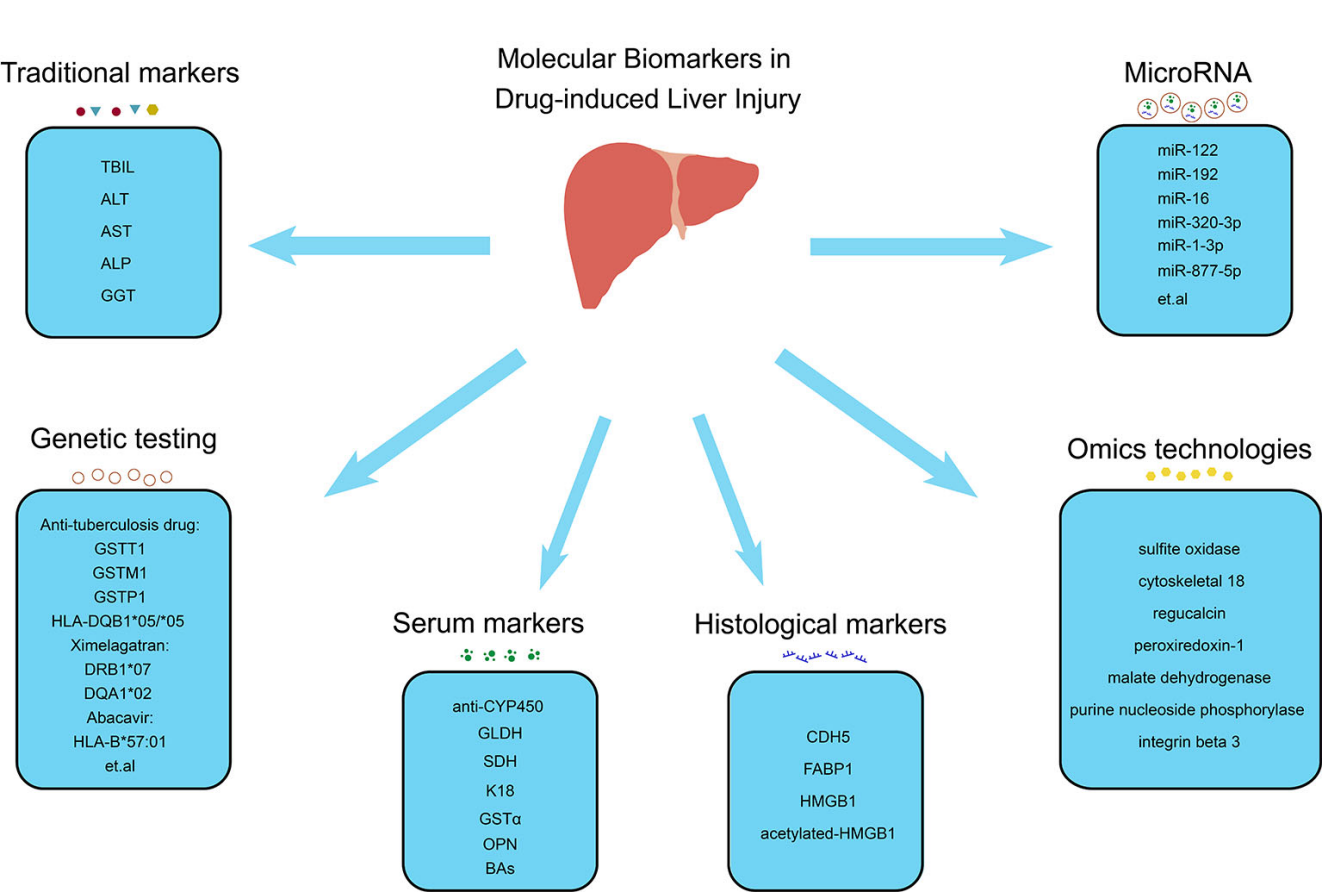
Molecular biomarkers in drug-induced liver injury. Molecular biomarkers of DILI include traditional markers (TBIL, ALT, AST, ALP and GGT), serum markers (anti-CYP450, GLDH, SDH, K18, GSTα, OPN and BAs), histological markers (CDH5, FABP1, HMGB1 and acetylated-HMGB1), microRNA (miR-122, miR-192, miR-6, miR-320-3p, miR-1-3p, miR-877-5p, etc.), genetic testing (GSTT1, GSTM1, GSTP1 and HLA-DQB*05/*05 associated with antituberculosis drugs, DRB1*07 and DQA1*02 associated with ximelagatran and HLA-B*57:01 associated with abacavir, etc.), and omics technologies (sulfite oxidase, cytoskeletal 18, regucalcin, peroxiredoxin-1, malate dehydrogenase, purine nucleoside phosphorylase, and integrin beta 3). TBIL, total bilirubin; ALT, alanine aminotransferase; AST aspartate aminotransferase; ALP, alkaline phosphatase; GGT, glutamyl transpeptidase; anti-CYP450, CYP450-antibodies; GLDH, glutamate dehydrogenase; SDH, sorbitol dehydrogenase; K18, keratin 18; GSTα, glutathione-S-transferase alpha; OPN, osteopontin; BAs, bile acids; CDH5, cadherin 5; FABP1, fatty acid binding protein 1; HMGB1, high mobility group box-1 protein.

DILI can mimic various other liver diseases, such as acute cholestatic hepatitis, acute hepatic necrosis, fatty liver disease, acute viral hepatitis-like syndrome, chronic hepatitis, and autoimmune hepatitis (Fontana et al., 2010). It can also cause illness, hospitalization, and even liver failure, requiring liver transplantation or even death. This can result in a considerable economic burden while also prevent many patients from experiencing the beneficial effect of certain drugs that have toxic adverse side effects in only a small number of people. Therefore, a standardized definition and diagnostic criteria should be developed for DILI. However, lacking effective biomarkers makes its diagnosis largely dependent on exclusion of alternative causes and a compatible drug history. Therefore, more effective biomarkers should better enable monitoring patients who receive suspected drugs, in order to reduce severe liver injury through early detection and subsequent cessation of medication. This review summarizes the current molecular biomarkers with the potential to provide more precise and accurate diagnosis and treatment of DILI (**Figure 1**).

## Overview of Current Molecular Biomarkers for DILI

### Traditional Biomarkers and Scoring Systems in DILI

Currently, due to the multifactorial character of DILI pathophysiology, its diagnosis is still based on exclusion of other causes that induce liver damage. Traditional testing of liver function relies on serum biomarkers such as alanine aminotransferase (ALT), aspartate aminotransferase (AST), alkaline phosphatase (ALP), glutamyl transpeptidase (GGT), and total bilirubin (TBIL). Elevations of TBIL levels correlate with whole liver function, while increase in AST/ALT concentrations reflects hepatocyte necrosis, and ALP levels reflect damage to biliary epithelial cells or canalicular membrane (Giannini et al., 2005; Padda et al., 2011). However, there are some drawbacks of relying solely on these traditional serum biomarkers as they are not entirely specific to liver injury, and do not provide a mechanistic understanding of injury patterns. ALT is considered to be more specific than AST, as it is primarily expressed in liver tissue and in low concentrations elsewhere, while AST can be presented in the liver, heart, skeletal muscle, kidney, brain, pancreas, and lung tissues, and even in white and red blood cells (Giboney, 2005). However, both ALT and AST have been shown to be elevated in the serum of individuals who performed extreme exercise or suffer from polymyositis (Nathwani et al., 2005). Further, ALP present in bone tissue was associated with osteoblast activity, while elevated ALP serum levels have also been reported in postmenopausal women, who also represent a high-risk population for DILI (Mukaiyama et al., 2015). These traditional biomarkers are released into the circulation following hepatocyte damage; however, they are unable to prognosticate early onset of DILI before overt liver injury occurs.

Currently, there are some scoring systems applied to diagnose DILI. The most popular of which include RUCAM, CDS, and DDW-J (the Digestive Disease Week Japan 2004) scale scores (**Table 1**). The RUCAM scale was developed at an international conference in 1993, and includes seven aspects: 1) time of the reaction onset, 2) course of the reaction, 3) risk factors for drug reaction, 4) concomitant drugs, 5) non-drug-related causes, 6) previous information on the drug, and 7) response to readministration (Danan and Benichou, 1993). The success of RUCAM is attributable to its objective, standardized, and liver-injury-specific approach (Danan and Teschke, 2018). The higher the calculated score, the greater the likelihood of DILI diagnosis. However, it has limitations in assessment method and arbitrary scoring, such as old age and alcohol use (Garcia-Cortes et al., 2011). According to the RUCAM system, patients over 55 years scores an extra point while recent data indicates that age is primarily related to the phenotype of DILI (Lucena et al., 2009; European Association for the Study of the Liver. Electronic address et al., 2019). Alcohol use scores another point; regular alcohol use may be a contributing factor for DILI associated with specific drugs (European Association for the Study of the Liver. Electronic address et al., 2019). But without distinguishing time, amount and duration of consumption, this may be not reasonable. Additionally, Maria et al. (Maria and Victorino, 1997) developed CDS in 1997, which provides improved feasibility of DILI diagnosis; however, it does not consider the pattern of liver injury. It includes five aspects: 1) temporal relationship between drug intake and the onset of clinical diagnosis, 2) exclusion of alternative causes, 3) extrahepatic manifestations, 4) intentional or accidental re-exposure to the drug, and 5) previous report that associated DILI with the drug (Maria and Victorino, 1997). Lastly, the DDW-J scale was developed by modifying the CIOMS (The Council for International Organizations of Medical Sciences)/RUCAM scale and includes eight aspects: 1) temporal relationship between drug intake and the onset of clinical diagnosis, 2) course of the reaction, 3) risk factors, 4) exclusion of other causes, 5) previous information, 6) extrahepatic manifestations of eosinophilia, 7) drug lymphocyte stimulation test, and 8) rechallenge (Watanabe and Shibuya, 2004; Tajiri and Shimizu, 2008; Hanatani et al., 2014). The latter two scoring systems do not exhibit better sensitivity or specificity than RUCAM, and thus, RUCAM continues to be the most commonly used method for DILI diagnosis. Nevertheless, there are still some limitations RUCAM has, and thus, exploring new molecular biomarkers may serve to improve its effectiveness in DILI diagnostics.

**Table 1:** Scoring Systems Applied to Diagnose DILI.

Scoring system	Scoring criteria (score)	Major biomarkers	Comments
**RUCAM** (Danan and Benichou, 1993; Garcia-Cortes et al., 2011; Danan and Teschke, 2018)	• Highly probable (> 8) • Probable (6–8) • Possible (3–5) • Unlikely (1–2) • Excluded (≤0)	• ALT • ALP	• It is an objective, standardized, and liver-injury-specific approach. • Its poor reliability and arbitrary scoring are questioned, such as alcohol use.
**CDS** (Maria and Victorino, 1997)	• Definite (> 17) • Probable (14–17) • Possible (10–13) • Unlikely (6–9) • Excluded (< 6)	• ALT • ALP	• It does not consider the pattern of liver injury, which often results in false diagnosis with cholestatic DILI.
**DDW-J** (Watanabe and Shibuya, 2004; Tajiri and Shimizu, 2008; Hanatani et al., 2014)	• Definite (≥5) • Probable (3–4) • Unlikely (≤2)	• ALT • ALP • Eosinophilic granulocyte	• Highly sensitive in Japanese patients; however, not evaluated in other populations.

RUCAM, Roussel Uclaf Causality Assessment Method; CDS, Clinical Diagnostic Scale; DDW-J, the Digestive Disease Week Japan 2004.

### Serum Biomarkers of DILI

At present, serum markers commonly used in DILI diagnosis include ALT, AST, GGT, ALP, and TBIL. However, many of these biomarkers are not specific to hepatotoxicity or do not provide information regarding the mechanism of the injury. In addition, biomarkers of hepatocyte injury, such as ALT and AST, enter the circulation after liver injury has already occurred; hence, they are unable to identify potential DILI cases before substantial liver injury has occurred. Although there are various biomarkers in clinical practice, their value in diagnosing and predicting DILI is limited. Therefore, it is important to explore new serological markers of DILI (**Table 2**).

**Table 2:** Serum Biomarkers of DILI.

**Serum biomarker**	**Advantage**	**Disadvantage**	**Comments**	**Reference**
**GLDH**	• Specific expression in liver. • Early recognition of DILI and not impacted by age, gender, or muscle injury. • Indicative of DILI prognosis.	• Elevated levels without hepatotoxicity. • Controversy in predicting hepatocyte necrosis.	• GLDH is related to DILI diagnosis and prognosis. • Possibly a specific biomarker for mitochondrial dysfunction.	Antoine et al., 2013; Schomaker et al., 2013; Flanigan et al., 2014; McGill et al., 2014; Singhal et al., 2014; Thulin et al., 2017
**K18**	• K18 and ccK18 ratio could predict necrosis and apoptosis. • Early recognition of DILI. • Not affected by muscle exercise. • Indicative of DILI prognosis.	• Not liver specific. • Also increased in other diseases.	• In various liver diseases, elevations in K18 and ccK18 may represent liver inflammation, and their ratio could assess the extent of hepatocyte necrosis and apoptosis.	Thulin et al., 2014; Ku et al., 2016; Chu et al., 2017; Wei et al., 2017; Sugimoto et al., 2018
**SDH**	• Early recognition of DILI could detect liver injury caused by special types of drugs. • Abundant in liver. • Short elimination half-life.	• No indicative of DILI prognosis.	• Previous studies have shown that SDH concentration was elevated in acute and mild liver injuries, which suggested that it may be a sensitive biomarker in liver inflammation.	Harrill et al., 2012; Metushi et al., 2012; Williams et al., 2012; Metushi et al., 2014b
**GST**	• GSTM1, GSTT1, and GSTP1 associated with ATDILI. • Increased GSTα in early DILI.	• Expensive and time-consuming genetic testing. • No reported in the prognosis of DILI.	• The genetic polymorphism of GST is closely related to ATDILI and GSTα presents its sensitivity in DILI.	Singla et al., 2014; Cai et al., 2015; Wu et al., 2016a; Sun et al., 2017; Tsai et al., 2017
BAs	• Informative about the mechanism of cholestasis caused by drugs.	• Lacking specificity in DILI.	• Elevated BA levels can be detected in various hepatobiliary diseases, which indicates its limited specificity in DILI.	Yamazaki et al., 2013; Qiu et al., 2016; Cepa et al., 2018
CYP450	• Participation in multiple drug metabolic reactions by CYP450 and its isoforms. • Poor outcome with anti-drug/anti-CYP P450 antibodies in antituberculosis drug-induced liver injury.	• Genetic testing is expensive and time-consuming. • The mechanism between drugs and CYP is complex and remained unknown.	• Treatment with some immune-related drugs may be effective once the role of immunity mechanism is determined.	Tang et al., 2013; Li et al., 2014; Metushi et al., 2014a; He et al., 2015; Metushi et al., 2016
**OPN**	• Prognosis of bad outcome in DILI. • Associated with the degree of liver necrosis.	• Not liver specific.	• OPN acts as a pro-inflammatory cytokine in inflammatory liver disease and attracts neutrophils, lymphocytes, and macrophages to hepatic injury sites.	Fan et al., 2015; Srungaram et al., 2015; Arriazu et al., 2017; Sampayo-Escobar et al., 2018

GLDH, glutamate dehydrogenase; K18, keratin 18; ccK18, caspase cleaves K18; SDH, sorbitol dehydrogenase; GST, glutathione S-transferase; GSTα, glutathione-S-transferase alpha; BAs, bile acids; CYP450, cytochrome P450; OPN, osteopontin.

#### Glutamate Dehydrogenase (GLDH)

GLDH, a mitochondrial protein encoded by the nuclear GLUD1 gene, is involved in amino acid oxidation and urea production (Schmidt and Schmidt, 1988; McDaniel, 1995). It is a relatively liver-specific enzyme expressed in the mitochondrial matrix of hepatocytes and is not altered in response to muscle injury when compared to ALT/AST (Schmidt and Schmidt, 1988; Flanigan et al., 2014). Because of its location in the cell, serum GLDH is a sensitive marker for liver disorders and reflects loss of mitochondrial integrity (Van Waes and Lieber, 1977; Singhal et al., 2014). Following onset of liver injury by administration of a single APAP high dose, GLDH levels are elevated rapidly and higher than those of ALT, and it is the better predictor in hepatocyte necrosis according to receiver operating characteristic curves, in contrast to ALT and miR-122 (Thulin et al., 2017). In addition, GLDH undergoes rapid elimination following APAP administration, while ALT remains elevated, indicating that GLDH may serve as a “real-time” monitor for active or persistent liver injury (Antoine et al., 2013; Schomaker et al., 2013). Alternatively, unlike ALT levels, serum GLDH activity has been reported to increase following APAP overdose as its levels were twofold higher in patients who died compared to those of survivors (McGill et al., 2012; McGill et al., 2014). Furthermore, levels of GLDH were stable between healthy and liver-injured subjects regardless of gender or age (Schomaker et al., 2013). However, it remains controversial whether GLDH can accurately predict hepatocyte necrosis, as necrosis may not result in mitochondrial toxicity, and it is a specific biomarker for mitochondrial dysfunction. This was supported by an experiment in which overdose of mice with furosemide caused hepatocyte necrosis, based on elevated ALT levels without increase of serum levels of GLDH and mitochondrial DNA (McGill et al., 2012). In addition, serum GLDH levels were found to be elevated without apparent hepatotoxicity, especially in healthy subjects who received heparin treatments (Harrill et al., 2012; Singhal et al., 2014). Therefore, the potential value of GLDH as a DILI biomarker is summarized in **Table 2**.

#### Keratin 18 (K18)

K18, a main type I intermediate filament cytoskeletal protein responsible for cell structure and integrity, was expressed by all single-layer epithelial cells, including hepatocytes and cholangiocytes (Thulin et al., 2014; Ku et al., 2016). At an early stage of apoptosis, caspase cleaves K18 form (ccK18) into a stable fragment and releases it into circulation (Caulin et al., 1997). The full-length variant of the protein is released from necrotic cells while ccK18 is derived from apoptotic cells (Thulin et al., 2014). Hence, serum proportion levels of K18 and ccK18 may represent markers of necrosis and apoptosis (Koelink et al., 2009). M65 and M30 are ELISA assays that detect circulating forms of K18 and ccK18, respectively. Furthermore, K18 is less affected by muscle movement than ALT or AST, with only 0.9-fold change observed in its expression after exercise in healthy volunteers, compared to 2.5- and 5.5-fold in ALT and AST, respectively (Thulin et al., 2014).

Liver injury can cause hepatocyte necrosis and apoptosis, depending on the severity of the liver injury (Neumann et al., 2014). Hence, early hepatocyte damage could be detected by measuring K18 and ccK18 levels (Galluzzi et al., 2012). At present, detection of K18 and ccK18 by immunoassays could be used as chemotherapy drug monitoring (Cummings et al., 2008). Further, serum M65 and M30 levels were significantly increased at hospital admission in patients with acetaminophen-induced ALF (AALF), compared to those of healthy controls. Additionally, poor outcome, liver transplantation, and death were correlated with elevated M65 and M30 levels upon admission (Possamai et al., 2013). Serum M30 levels were associated with liver dysfunction and post-transplant graft failure, with levels >900 U/L indicating 1-year graft loss (Possamai et al., 2013). A recent study demonstrated that serum fragment K18 was higher in severe idiosyncratic DILI as well (Xie et al., 2019). However, in end stage of AALF, ccK18 levels decreased throughout the liver while it persisted in circulation at levels exceeding those in healthy controls, suggesting that ccK18 is not of hepatic origin (Possamai et al., 2013). Other diseases were also associated with elevated M30 and M65 levels, such as non-small-cell lung cancer, biliary tract cancer, chronic hepatitis B (CHB), hepatitis C virus (HCV), and nonalcoholic fatty liver disease (NAFLD) (Kronenberger et al., 2005; Joka et al., 2012; Chu et al., 2017; Wei et al., 2017; Sugimoto et al., 2018). Therefore, K18 may not be liver specific as it is expressed by all the single-epithelial cells. Nevertheless, various liver diseases cause elevated K18 and ccK18 levels, which is representative of liver inflammation, and their ratio could be used to assess the extent of hepatocyte necrosis and apoptosis (**Table 2**).

#### Sorbitol Dehydrogenase (SDH)

SDH, a cytoplasmic enzyme, is found predominantly in the liver and testis, and has been used as a diagnostic marker of hepatic and testis diseases for many years (Asada and Galambos, 1963; Hodgen and Sherins, 1973). It is a sensitive indicator of hepatocellular damage because of its substantively higher levels in the liver compared to other traditional serum markers (Hodgen and Sherins, 1973). In healthy subjects who received subcutaneous injections of unfractionated heparin, elevated serum SDH levels were observed without clinical symptoms, suggesting that SDH could detect mild liver dysfunction (Harrill et al., 2012). Certain drugs have been reported to inhibit the expression of ALT and mask the real liver injury. For example, low doses of D-penicillamine (10 or 15 mg/day) caused a slight increase in SDH and GLDH activities before histopathological changes, while no increase in ALT concentration was observed in D-penicillamine-induced granulomatous hepatitis in mice (Metushi et al., 2014b). Moreover, isoniazid (INH) inhibited the ALT assay by directly reacting with the aldehyde group of pyridoxal 5´ phosphate. However, SDH was a better biomarker in INH-related liver injury (Metushi et al., 2012). Previous studies have shown that SDH concentration was elevated in acute and mild liver injuries, suggesting that it may be a sensitive biomarker for liver inflammation (**Table 2**).

#### Glutathione S-Transferase (GST)

GST is a cytosolic enzyme abundant in the liver and serves as a biomarker of liver hepatotoxicity; it is released into the plasma from liver cytosol following impaired liver function (Yamamoto and Yamada, 2016; Zhang et al., 2017). Hence, monitoring the GST activity level may be a potential strategy for the diagnosis of DILI. Similarly, GST is an important drug metabolizing enzyme, and gene polymorphisms in GST have been associated with susceptibility to DILI. Specifically, GSTM1 and GSTT1 double null genotypes may be risk factors for developing antituberculosis drug-related hepatotoxicity in Indian patients (Singla et al., 2014). However, several studies have shown that GSTM1 null genotypes, and not GSTT1 genotype, are related to high risk of antituberculosis drug-related liver injury (ATDILI) (Sun et al., 2008; Cai et al., 2015; Yang et al., 2019). Additionally, GSTP1 has been reported to influence the risk of ATDILI (Wu et al., 2016a).

Glutathione-S-transferase alpha (GSTα) is an isozyme of GST, and a viable biomarker as its levels increase more significantly than those of AST or ALT during hepatotoxicity (Giffen et al., 2002). Additionally, elevated GSTα was correlated with hepatic injury (Giffen et al., 2002). GSTα also showed enhanced specificity in the detection of liver injury and hepatocyte vacuolation, reducing false positive results based on ALT levels in extrahepatic tissues (Bailey et al., 2012). Further, GST genetic polymorphisms were associated with various liver diseases. Specifically, in patients with acute-on-chronic hepatitis B liver failure (ACLF), GSTM3 gene promoter methylation levels were significantly elevated compared to those in patients with chronic hepatitis B and healthy controls, and levels of GSTM3 methylation were associated with prognosis of ACLF (Sun et al., 2017). In NAFLD, GST plasma concentration was associated with hepatic lipid deposition (Tsai et al., 2017). Finally, the genetic polymorphism of GST has been reported to be closely related to ATDILI, and GSTα has presented its sensitivity in DILI (**Table 2**).

#### Bile Acids (BAs)

BAs are synthesized in the liver from cholesterol and are secreted into the bile canaliculi through the canalicular membrane, a process driven by the bile salt export pump (BSEP) (Schadt et al., 2016). BAs are biomarkers of DILI due to their amphiphilic and strong emulsifying detergent properties, which may damage the integrity of cell membrane and result in cytotoxic effects. The accumulation of BAs in hepatocytes can trigger adaptive regulatory mechanisms, including inhibition of cholesterol oxidation and upregulation in expression of the BA transporter protein to prevent cellular damage. Therefore, the toxic effects of BAs could be exacerbated once some drugs break the BA regulatory pathway (Qiu et al., 2016). Moreover, drug-induced cholestasis has been correlated with inhibition of BSEP. Specifically, cyclosporine A, rifampicin, bosentan, troglitazone, and various other drugs were reported to be potent BSEP inhibitors (Pauli-Magnus et al., 2005). Additionally, the newly reported drug fasiglifam, developed for the treatment of type 2 diabetes, was voluntarily terminated in Phase III trials due to adverse liver effects caused by inhibition of BA transporters (Longo et al., 2019). Previous study detected elevated glycocholate, taurocholate, and urinary cholate levels before observable changes in serum TBIL and liver enzymes occurred in rats, suggesting that these metabolites could serve as biomarkers to improve DILI detection in the preclinical stage (Yamazaki et al., 2013). This phenomenon was also noted by a recent study that showed that BA fluctuation in levels was already evident at early stages after low-dose methapyrilene-induced liver injury (Cepa et al., 2018). Similarly, elevations of conjugated BAs were observed in children with APAP overdose compared to healthy controls (James et al., 2015). Nevertheless, elevated BA levels can be detected in various hepatobiliary diseases, which indicates its limited specificity in DILI (**Table 2**).

#### Cytochrome P450 (CYP450)

CYP450 is a superfamily of drug metabolizing enzymes that are localized in the hepatocyte endoplasmic reticula. Certain drugs and their metabolites may cause liver damage by affecting CYP450 or its isoforms. Specifically, bortezomib was reported to decrease hepatocyte nuclear factor−1α-induced promoter activation of cytochrome P450 2E1 (CYP2E1) and induced endoplasmic reticulum stress, thereby significantly down-regulating CYP2E1 expression and alleviating APAP-, CCl_4_-, and thioacetamide-induced liver injury in mice (Park et al., 2016). In addition, certain cytochrome enzymes, including CYP2E1, CYP2B6, and CYP3A4, were associated with the mechanism responsible for causing increased hepatic necrosis (Li et al., 2014). Further, since the occurrence of DILI has been related to genetic vulnerability, many studies have focused on single nucleotide polymorphisms (SNP) in transporter genes (Au et al., 2011). A potential correlation was identified between wild-type CYP2E1 genotype and increased susceptibility to liver injury following treatment of antituberculosis (Sun et al., 2008). But there is a controversy about the existence of significant correlations between polymorphisms of CYP1A1, CYP3A4, CYP2C9, and CYP2C19 and the risk of ATDILI; only a few polymorphisms were associated with hepatotoxicity (Tang et al., 2013; He et al., 2015).

Several drugs that induce liver injury were associated with the presence of serum autoantibodies. Patients suffering from isoniazid-induced hepatotoxicity presented cytochrome antibodies, as a difference to patients with only mild INH-induced liver injury (Metushi et al., 2014a). Furthermore, although the immune system plays a critical role in INH-induced hepatotoxicity, immune tolerance is often achieved with mild liver injury, while other cases experience poor outcomes with anti-drug/anti-CYP450, often resulting in severe liver failure even following termination of drug treatment (Metushi et al., 2016). Therefore, administration of specific immunomodulatory drugs may prove effective, leading to improved clinical outcomes, should the role of immunity in the mechanism of liver failure be better characterized (**Table 2**).

#### Osteopontin (OPN)

OPN is a secreted multifunctional protein, the expression of which is regulated by interleukin-1β and other acute inflammatory mediators (Serlin et al., 2006; Kahles et al., 2014). OPN is secreted by a myriad of cells and tissues, including macrophages, smooth muscle cells, epithelial cells, and endothelial cells (Sampayo-Escobar et al., 2018). Moreover, OPN levels are associated with the degree of liver necrosis in acute liver injury; however, its involvement in preventing the progression of liver disease requires further investigation (Arai et al., 2006; Srungaram et al., 2015). In mice, OPN deficiency served to restrict neutrophil infiltration and macrophage accumulation and inhibited the release of pro-inflammatory cytokines (He et al., 2012). Similarly, by blocking OPN expression, the sensitivity to acetaminophen was reduced and mice developed the capacity to survive a drug overdose (Arai et al., 2006; He et al., 2012). However, another study evidenced that OPN improved the survival of hepatocytes in diethylnitrosamine-induced liver injury *via* inhibition of NF-κB activation and reduced production of inflammatory mediators (Fan et al., 2015). Still further, a recent study observed that OPN upregulated the acetylation and translocation of HMGB1 (high mobility group box-1 protein) in hepatic stellate cells, which then promoted the production of collagen-I. This finding suggests a novel therapeutic method for targeting OPN-HMGB1 signaling pathways (Arriazu et al., 2017). Also, reduced plasma OPN levels were observed in patients with ALF who either died or received liver transplants, compared to those of patients with better outcomes (Srungaram et al., 2015). Finally, the Drug-Induced Liver Injury Network reported that elevated OPN, K18, and macrophage colony-stimulating factor receptor levels served as predictors of death or transplantation in DILI. Taken together these studies suggest that OPN acts as a pro-inflammatory cytokine in inflammatory liver disease and attracts neutrophils, lymphocytes, and macrophages to hepatic injury sites (**Table 2**).

### Histological Markers of DILI

The histological features and diagnosis of DILI, partially indicated by liver biopsy, were summarized in the European Association for the Study of the Liver guidelines for DILI in 2018. Mild or moderate liver injury is characterized by granulomas and eosinophilic infiltration, while poor outcomes, including severe liver injury, liver transplantation, or death, present neutrophil infiltration, higher degree of necrosis and fibrosis, cholangiolar cholestasis, ductular reaction, portal venopathy, and microvesicular steatosis (European Association for the Study of the Liver. Electronic address et al., 2019). Currently, several histological biomarkers were associated with diagnosis and prognosis of DILI (**Table 3**).

**Table 3:** Histological Biomarkers of DILI.

**Histological biomarker**	**Advantage**	**Disadvantage**	**Comments**	**Reference**
**HMGB1**	• Early recognition of DILI. • Involved in DILI prognosis.	• Not liver specific.	• HMGB1 acts as a mediator playing a key role both in the early and late stages of systemic inflammation and its acetylated form may be a better biomarker for prognosis prediction in DILI.	Wang et al., 2013; Nakamura et al., 2014; Huebener et al., 2015; Lundback et al., 2016
**FABP1**	• Abundant in liver and weak expression in heart and skeletal muscles. • Elevations in FABP1 levels was associated with bad DILI outcome.	• Elevated in various liver diseases.	• FABP1 has superior characteristics regarding tissue distribution and kinetics compared to ALT.	Wu et al., 2016b; Karvellas et al., 2017; Mikus et al., 2017; Wu et al., 2017
**CDH5**	• Elevated in DILI and sinusoidal dilatation.	• Not liver specific.	• At present, there are a few reports about the role of CDH5 in DILI, so it needs further study and confirmation.	Mikus et al., 2017; Jarzabek et al., 2018

HMGB1, high mobility group box-1 protein; FABP1, fatty acid binding protein 1; CDH5, cadherin 5.

#### High Mobility Group Box-1 Protein (HMGB1)

HMGB1 was originally discovered as a nuclear protein present in most tissues and primarily involved in DNA replication, recombination, repair, and gene transcriptional regulation. It becomes released into the extracellular space following cellular damage (Rovere-Querini et al., 2004; Huebener et al., 2015). Necrotic and immune cells are associated with HMGB1 (Gardella et al., 2002; Semino et al., 2005). In normal conditions, non-acetylated and thiol forms of HMGB1 are located in the nucleus, released after cell damage, and converted to disulfide form. In addition, acetylated-HMGB1 can be secreted by immune cells and readily oxidized in the extracellular space (Venereau et al., 2016). When released into the extracellular space, HMGB1 can bind to the receptors of advanced glycation end products and/or toll-like receptors, which eventually leads to a pro-inflammatory positive feedback network, recruiting and activating more inflammatory cells to the site of injury (Fiuza et al., 2003; Palumbo et al., 2004; Venereau et al., 2016). During APAP hepatotoxicity, serum HMGB1 levels become significantly increased during early stages compared to those of ALT; however, they were found to return to baseline levels more quickly once liver function was improved (Antoine et al., 2009). Additionally, acetylated HMGB1 levels only increased in patients with a poor outcome following development of DILI induced by APAP (Antoine et al., 2012). Therefore, acetylated HMGB1 may be a prognostic indicator for DILI.

Moreover, inhibition of HMGB1 expression may also be an effective approach for DILI treatment. Several therapeutic methods have been used in APAP-induced ALF, including anti-HMGB1 antibodies, chemical interventions, and naturally derived compounds, which have been used to alleviate the release of HMGB1 and prevent pro-inflammatory reactions (Dragomir et al., 2011; Wang et al., 2013; Lundback et al., 2016). When compared to traditional treatment of N-acetylcysteine, treatment with humanized HMGB1-neutralizing antibodies was more effective and provided a prolonged therapeutic window (Lundback et al., 2016). For example, recombinant human soluble thrombomodulin (rTM) suppressed the expression of serum HMGB1 in monocrotaline-induced sinusoidal obstruction syndrome; thus, these results indicated that rTM decreased circulatory HMGB1 and inhibited active neutrophil accumulation, with significantly reduced serum ALT levels and an improved patient outcome (Nakamura et al., 2014). Hence, HMGB1 acts as a mediator playing a key role both in the early and late stages of systemic inflammation and its acetylated form may be a better biomarker for prognosis prediction in DILI (**Table 3**). Recently, many novel HMGB1-targeted treatments have been developed, which should be considered for future clinical treatment.

#### Fatty Acid Binding Protein 1 (FABP1)

FABP1 is abundantly expressed in the cytosol of liver parenchymal cells and associated with metabolism, storage, transport, and utilization of fatty acids (Bass, 1988). A cohort study collected 1,196 samples from 241 individuals using anti-FABP1 immunohistochemistry analysis, and results showed that FABP1 is primarily expressed in the liver and kidneys, and only weakly expressed in the heart and skeletal muscles (Mikus et al., 2017). In addition, FABP1 is a promising indicator of ongoing liver injury as its levels were found to increase before those of ALT, and decreased after treatment stopped, while ALT levels remained unchanged (Mikus et al., 2017). Further, APAP-induced ALF survivors had significantly lower serum FABP1 levels than did non-survivors in early stage and late stage, indicating the elevated FABP1 levels in patients with APAP-induced ALF were associated with a poor outcome (Karvellas et al., 2017). Furthermore, a higher FABP1 concentration (> 350 ng/ml) was associated with increased mortality (Karvellas et al., 2017). Increased FABP1 levels were also observed in CHB and NAFLD, while reduced expression served to effectively protect against hepatocyte steatosis and injury, providing a potential treatment strategy for liver diseases (Wu et al., 2016b; Wu et al., 2017). FABP1 offers superior characteristics regarding tissue distribution and kinetics compared to ALT (**Table 3**), and it is involved in numerous metabolic disease processes, including those of liver disease, cancer, diabetes, obesity, and atherosclerosis. Additionally, by inhibiting its expression, new insights into effective DILI treatment strategies may be obtained.

#### Cadherin 5 (CDH5)

CDH5, also called VE-cadherin, is one of calcium-dependent cell adhesion proteins, and is an important component of endothelial adherent junctions. CDH5 can be found in most tissues, save for bone marrow, with placenta, lung, and adipose tissues being the highest ranked. Further, its increased concentration was reported in APAP-induced liver injury and oxaliplatin-induced sinusoidal dilatation (Mikus et al., 2017; Jarzabek et al., 2018). Currently, few studies have reported on the role of CDH5 in DILI; therefore, this should serve as an important focus for future investigations (**Table 3**).

### MicroRNA-Related Biomarkers

MicroRNAs (miRNAs) are small noncoding RNAs, primarily involved in post-transcriptional gene regulation and are relatively stable in biofluids. Changes in the expression of miRNAs were involved in a variety of pathophysiological events, including liver injury (Kagawa et al., 2018). Variants of miR-122 accounted for approximately 72% of the total liver miRNA, with superior characteristics regarding liver distribution (Lagos-Quintana et al., 2002). It is a specific liver biomarker as demonstrated in a cohort study of subjects who experienced extreme exercise-induced muscular injury. The research showed that levels of miR-122 were only increased by 0.3-fold after exercise, while those of ALT/AST experienced 2.5- and 5.5-fold changes, respectively (Thulin et al., 2014). Furthermore, in mice, upregulation of miR-122 and miR-192 levels was earlier than ALT in APAP-DILI, and their plasma levels were found to correlate with the histopathology of liver degeneration (Wang et al., 2009). Additionally, miR-122 was approximately 30-fold above the median concentration in tolvaptan-induced liver injury with no obvious hepatocyte necrosis, indicating its sensitivity for the detection of liver dysfunction at an early stage (Mosedale et al., 2018). Another study confirmed this phenomenon by showing that tissue miR-122 levels were significantly decreased in early stages with gradual increase of miR-155; and the ratio of miR-122/155 was highly associated with hepatotoxicity in INH-induced liver injury compared to the ratio of ALT/AST (Song et al., 2016). Moreover, miR-122 and miR-192, miR-194, miR-483, and miR-210 were identified as liver-enriched APAP overdose-responsive miRNAs (Krauskopf et al., 2015). MicroRNAs were also associated with the prognosis of DILI. Specifically, decreased serum levels of miR-122, miR-4463, and miR-4270 were correlated with death within 6 months, while a combination of decreased serum albumin and miR-122 provided accurately predictive values for survival of 6 months (Russo et al., 2017). It is convenient to detect miR-122 in capillary blood obtained by finger venipuncture as its levels were associated with the plasma and venous whole blood concentration (Vliegenthart et al., 2017). These results clearly demonstrated the usefulness of miR-122 as a diagnostic and predictive marker of DILI.

The role of miRNAs in liver disease was also correlated with immune inflammatory responses. It has been reported that miR-16 down-regulated levels of the anti-apoptotic protein, Bcl2, as well as the expression of its gene, while activating TNF-mediated apoptosis through targeting caspase-8 during hepatocyte apoptosis (Szabo and Bala, 2013). Thus, miRNAs may aggravate hepatocyte injury by triggering apoptosis signaling in DILI. In fact, elevation in miR-877-5p and phosphoenolpyruvate carboxykinase 1 (PEPCK) expression levels was observed after trovafloxacin administration. Moreover, PEPCK was correlated with development of apoptotic cell death and its expression was associated with miR-877-5p expression levels in trovafloxacin-induced liver injury (Mitsugi et al., 2016). Other microRNAs also play a crucial role in DILI. Elevated miR-1-3p and let-7b-5p levels precede traditional biomarkers in hepatocellular injury; miR-218a-5p and miR-143-3p were increased in early stage of cholestasis; and increased miR-320-3p and decreased miR-1-3p levels were associated with early stages of hepatic steatosis in rats (Kagawa et al., 2018). The biological significance and utility of miRNAs in liver disease is a rapidly growing field, and these miRNAs may be particularly important in DILI.

### DILI-Related Genetic Testing

Genetic testing has become effective due to the associations reported between human leucocyte antigen (HLA) and certain drugs that cause idiosyncratic DILI. Many of the HLA alleles associated with DILI have highly negative predictive values; hence, genotyping may prove useful in the exclusion of hepatotoxicity due to certain particular drugs, or in identifying the causative drugs when the patient is taking more than one hepatotoxic agent (Aithal, 2015). In addition, an extensive overlap exists among the risk alleles associated with clinical toxicity profiles, owing to structurally dissimilar compounds. For instance, DRB1*07:01 is a risk allele for flucloxacillin, ximelagatran, and lapatinib-related DILI, while it serves a protective role in amoxicillin-clavulanate related liver injury. Similarly, DRB1*15:01 was correlated with DILI secondary to amoxicillin-clavulanate and lumiracoxib, yet was associated with reduced risk of flucloxacillin-related DILI (Donaldson et al., 2010). The correlation between HLA and DILI indicated that the adaptive immune system may play a key role in the disease etiology. Regarding flucloxacillin, HLA-B*57:01 restricted activation of drug-specific T cells was found in flucloxacillin-induced liver injury (Monshi et al., 2013). Abacavir, an HIV therapeutic drug, has been associated with HLA-B*57:01 allele; studies showed that abacavir induced loading of novel self-peptides onto HLA-B*57:01, which elicited an autoimmune response of polyclonal T cells and multiple organ system toxicity (Norcross et al., 2012).

Recently, genome-wide association studies (GWASs) demonstrated that HLA-A*33:01 was a risk factor for cholestatic or mixed DILI, save for hepatocellular DILI caused by terbinafine and possibly fenofibrate or ticlopidine (Nicoletti et al., 2017). Moreover, patients carrying the DRB1*07 and DQA1*02 alleles during the long-term treatment with the oral direct thrombin inhibitor ximelagatran were more likely to have elevated ALT levels (Kindmark et al., 2008). Another study, including 89 cases with ATDILI, found that subjects with HLA-DQB1*05/*05 genotype were more likely to develop liver injury (Chen et al., 2015). Since it is difficult to discriminate DILI and autoimmune hepatitis (AIH) triggered by drugs in a clinical setting, thus early diagnosis and proper management are critical in both diseases or severe acute or chronic outcomes may result (Suzuki et al., 2011). Although liver biopsies are generally the most effective way to discriminate between those conditions, it is an invasive procedure and carries with it certain inherent risks; therefore, genetic testing may be a better choice. Additionally, HLA-DRB1*03:01 and HLA-DRB1*04:01 are risk factors for AIH type 1, which may provide a reference for the differential diagnosis between DILI and AIH (de Boer et al., 2014). However, the high cost associated with genetic testing makes its use limited. Nevertheless, genetic testing can be used to rule out DILI caused by specific drugs and may, therefore, become a routine auxiliary examination method.

### Omics Technologies in DILI

The development of omics technologies provides a novel, effective method for research and exploration of biomarkers in DILI. One proteomics study investigating the protective effect of dioscin against APAP-DILI validated six differentially expressed proteins (sulfite oxidase, cytoskeletal 18, regucalcin, peroxiredoxin-1, malate dehydrogenase, and purine nucleoside phosphorylase) involved in the hepatoprotective effect of dioscin by using proteomics (Zhao et al., 2012). Results showed that APAP causes mitochondrial damage by interfering with the metabolism of these proteins, but dioscin exhibited a remarkable protective effect against APAP-DILI (Zhao et al., 2012). Another study using proteomics analysis identified over 2,700 proteins differentially regulated in monocyte-derived hepatocyte-like cells derived from individual patients with Diclofenac-DILI. In addition, this study showed that integrin beta 3 (ITGB3) can be as readily detected as liver enzymes and is able to identify the cause of liver injury (Dragoi et al., 2018). Hence, omics-based research can offer information on the potential mechanisms of DILI and promote the identification of early diagnostic biomarkers; however, the major constraint of this technology is that, to date, it has been performed primarily on intrinsic DILI in basic experiment research.

## Discussion

### Future Prospects and Studies

DILI will remain a major public health challenge in the coming years due to the increasing use of herbal and dietary supplements in an aging and overweight population. According to a recent study, new molecular biomarkers have revealed better sensitivity and specificity compared with ALT and AST. Specifically, GLDH and miR-122 were found to be more readily detectable as biomarkers of APAP-DILI (Thulin et al., 2017). Further, the combined model of miR-122, HMGB1, and K18 more accurately predicted liver injury than did ALT alone in APAP-DILI (Dear et al., 2018). The sensitivity of these biomarkers (such as GLDH, K18, HMGB1, FABP1, and miR-122) to detect DILI is reflected in their rapid elevation compared to ALT or AST, and subsequent rapid elimination following termination of drug use, while traditional biomarkers remain elevated. Elevated ALT levels are generally believed to be liver specific; however, it also occurs following excessive exercise and muscle diseases. Alternatively, GLDH and miR-122 are abundant in liver, yet are not influenced by muscle status, which may make up for the deficiency of ALT to detect liver injury. Hence, GLDH, K18, HMGB1, FABP1, and miR-122 levels provide a certain value in predicting the prognosis of DILI. In susceptible populations, drug-related genetic testing can be employed to avoid severe liver injury. These studies clearly demonstrate the specificity of new biomarkers in DILI compared to traditional biomarkers.

Although traditional indicators lack sensitivity and specificity, they have, nevertheless, served a critical role in recent years. However, the addition of new molecular biomarkers could improve DILI detection, prediction, and risk assessment. Ideal biomarkers may be related to different drug types and disease types; however, as of yet, few studies have examined these relationships. Currently, many of the studies examining biomarkers in DILI are focused on APAP and antituberculosis drugs. APAP-induced liver injury found elevated GLDH, K18, HMGB1, FABP1, CDH5, and miR-122 (Antoine et al., 2009; Wang et al., 2009; Antoine et al., 2013; Possamai et al., 2013; Karvellas et al., 2017; Mikus et al., 2017). GLDH may be a specific biomarker for mitochondrial dysfunction. The ratio of K18 and ccK18 may assess the extent of hepatocyte necrosis and apoptosis. HMGM1, OPN, and miR-122 could act as inflammatory factors in APAP-related liver injury. Further, antituberculosis DILI was found to be associated with an increase in SDH, BAs, and anti-CYP450 antibodies and patients with GSTM1, GSTP1 or HLA-DQB1*05/*05 genotypes are more likely to develop liver injury (Pauli-Magnus et al., 2005; Metushi et al., 2012; Cai et al., 2015; Chen et al., 2015; Metushi et al., 2016; Wu et al., 2016a; Sun et al., 2017). Moreover, SDH was elevated in acute and mild liver injuries and BAs have been detected in various hepatobiliary diseases. However, current studies have not suggested that these biomarkers are related to a particular type of drug. Future investigations should also examine the use of certain drugs in high risk populations to detect early liver injury, in an attempt to provide appropriate treatment and prevent disease progression. The current DILI studies are largely concentrated in preclinical research, with clinical transformation requiring significantly more time to extensive analysis to complete. Nevertheless, we have summarized the biomarkers of DILI in clinical and animal studies (**Table 4**).

**Table 4:** Studies of Clinical and Basic Studying Data in Biomarkers.

**Biomarker**	**Clinical data**	**Basic studying data**
**GLDH**	McGill et al., 2012; Harrill et al., 2012; Antoine et al., 2013; Schomaker et al., 2013; McGill et al., 2014; Singhal et al., 2014	-
**K18**	Koelink et al., 2009; Possamai et al., 2013; Thulin et al., 2014; Xie et al., 2019	Cummings et al., 2008
**SDH**	Harrill et al., 2012	Metushi et al., 2012; Metushi et al., 2014b
**GST**	Sun et al., 2008; Singla et al., 2014; Cai et al., 2015; Wu et al., 2016a; Sun et al., 2017; Yang et al., 2019	Giffen et al., 2002; Bailey et al., 2012; Tsai et al., 2017
**BAs**	James et al., 2015; Longo et al., 2019	Yamazaki et al., 2013; Qiu et al., 2016; Cepa et al., 2018
**CYP450**	Sun et al., 2008; Tang et al., 2013; Tang et al., 2013; Metushi et al., 2014a; He et al., 2015	Li et al., 2014; Park et al., 2016
**OPN**	Arai et al., 2006; Srungaram et al., 2015	He et al., 2012; Fan et al., 2015; Arriazu et al., 2017
**HMGB1**	Antoine et al., 2012	Rovere-Querini et al., 2004; Antoine et al., 2009; Dragomir et al., 2011; Wang et al., 2013; Nakamura et al., 2014; Huebener et al., 2015; Lundback et al., 2016
**FABP1**	Mikus et al., 2017; Karvellas et al., 2017	-
**CDH5**	Mikus et al., 2017; Jarzabek et al., 2018	-
**miRNAs**	Thulin et al., 2014; Krauskopf et al., 2015; Russo et al., 2017; Vliegenthart et al., 2017; Mosedale et al., 2018	Wang et al., 2009; Song et al., 2016; Mitsugi et al., 2016; Kagawa et al., 2018
**DILI-related genetic testing**	Kindmark et al., 2008; Donaldson et al., 2010; Suzuki et al., 2011; Norcross et al., 2012; Monshi et al., 2013; de Boer et al., 2014; Chen et al., 2015; Nicoletti et al., 2017	-

GLDH, glutamate dehydrogenase; K18, keratin 18; ccK18, caspase cleaves K18; SDH, sorbitol dehydrogenase; GST, glutathione S-transferase; GSTα, Glutathione-S-transferase alpha; BAs, Bile acids; CYP450, Cytochrome P450; OPN, osteopontin. HMGB1, high mobility group box-1 protein; FABP1, fatty acid binding protein 1; CDH5, cadherin 5.

TCM are becoming more popular worldwide with an increased preference for herb use. Research shows that following treatment with Fructus Meliae Toosendan (a known hepatotoxic TCM), 8 miRNAs and 1,723 mRNAs were significantly differentially expressed (Zheng et al., 2015). Other studies found that miRNA374a, tumor necrosis factor receptor, and exosomes were related to the mechanism of inflammatory bowel disease, and recent evidence supports that hepatocyte-derived exosomes play a key role in the pathogenesis of DILI (Zhou et al., 2017; Thacker et al., 2018; Xiong et al., 2018; Zhang et al., 2019). These studies provide new insights into these potential biomarkers for TCM-induced liver injury. However, many obstacles must be overcome prior to the implementation of these biomarkers in clinical practice. For instance, our current knowledge regarding these biomarkers is derived from relatively limited patient populations, and research has not yet delved into the specific DILI mechanism or different biochemical patterns of the liver injury. Additionally, it is impossible to assess the efficacy of these biomarkers as predictors of DILI, unless genetic testing is first performed. Another noteworthy issue is that current biomarkers are not specific to DILI; various liver injuries cause an increase in the levels of these biomarkers, while only genetic testing possesses high specificity for DILI. However, the high associated cost and time requirements of genetic testing may limit its use. Therefore, it is necessary to explore new and convenient detection methods and conduct large sample size studies.

A recent study evidenced that adaptive immune response played a key role in the mechanism of idiosyncratic DILI, and in most instances, chemical properties of drugs and the formation of active metabolites act as haptens; this can lead to lethal consequences or induce immune adaptive responses that dampen these processes (European Association for the Study of the Liver. Electronic address et al., 2019). Besides, suspicious drugs induced liver injury in clinical practice can be identified through the online databases: 1) https://www.ncbi.nlm.nih.gov and 2) http://www.hepatox.org/. Further investigations should include: 1) identifying the role of new molecular biomarkers in the mechanism of DILI, 2) assessing the performance of these biomarkers in DILI compared to other liver diseases, and 3) comparing these biomarkers in liver injury caused by the same drug dosage. Therefore, the comprehensive approach is to combine new biomarkers with traditional biomarkers and DILI-related scoring systems, which can provide potentially enhanced value for the diagnosis, treatment, and prognosis of DILI.

## Author Contributions

TZ and CJ conceived and designed the project. Each author has contributed significantly to the submitted work. SF and DW drafted the manuscript. SF, DW, WJ, JLi, and JLo revised the manuscript. All authors read and approved the final manuscript.

## Funding

This work was supported by Post-Doctor Research Project, West China Hospital, Sichuan University (grant number 2018HXBH005).

## Conflict of Interest

The authors declare that the research was conducted in the absence of any commercial or financial relationships that could be construed as a potential conflict of interest.
